# Laser surgery of zebrafish (*Danio rerio*) embryos using femtosecond laser pulses: Optimal parameters for exogenous material delivery, and the laser's effect on short- and long-term development

**DOI:** 10.1186/1472-6750-8-7

**Published:** 2008-01-29

**Authors:** Vikram Kohli, Abdulhakem Y Elezzabi

**Affiliations:** 1 9107-116 St, Ultrafast Photonics and Nano-Optics Laboratory, Department of Electrical and Computer Engineering, University of Alberta, Edmonton, T6G2V4, Canada

## Abstract

**Background:**

Femtosecond (fs) laser pulses have recently received wide interest as an alternative tool for manipulating living biological systems. In various model organisms the excision of cellular components and the intracellular delivery of foreign exogenous materials have been reported. However, the effect of the applied fs laser pulses on cell viability and development has yet to be determined. Using the zebrafish (*Danio rerio*) as our animal model system, we address both the short- and long-term developmental changes following laser surgery on zebrafish embryonic cells.

**Results:**

An exogenous fluorescent probe, fluorescein isothiocyanate (FITC), was successfully introduced into blastomere cells and found to diffuse throughout all developing cells. Using the reported manipulation tool, we addressed whether the applied fs laser pulses induced any short- or long-term developmental effects in embryos reared to 2 and 7 days post-fertilization (dpf). Using light microscopy and scanning electron microscopy we compared key developmental features of laser-manipulated and control samples, including the olfactory pit, dorsal, ventral and pectoral fins, notochord, pectoral fin buds, otic capsule, otic vesicle, neuromast patterning, and kinocilia of the olfactory pit rim and cristae of the lateral wall of the ear.

**Conclusion:**

In our study, no significant differences in hatching rates and developmental morphologies were observed in laser-manipulated samples relative to controls. This tool represents an effective non-destructive technique for potential medical and biological applications.

## Background

The ability to non-invasively manipulate cells and multicellular biological systems is important for developments in tissue engineering, cell-based therapeutics and molecular and developmental biology. Of particular interest is the manipulation of zebrafish (*Danio rerio*) embryos, an aquatic vertebrate organism that is genetically and developmentally closer to humans than the common invertebrates *Drosophila melanogaster *and *Caenorhabditis elegans *[1,2].

The zebrafish is an attractive model system that has received wide attention among vertebrate embryologists, zoologists and developmental biologists. Important features that distinguish this model system from others are: the transparency of the developing embryos within the proteinaceous membrane (chorion), the progression of embryogenesis where the formation of organs can be clearly observed, and the occurrence of sexual maturity within 2 to 3 months [3]. Presently, zebrafish are used in the study of genetics, drug monitoring, human disease, cardiac function and blood disorders [4-10]. Despite these benefits, non-invasive intracellular manipulation of embryos has been challenging, owing to the proteinaceous chorion, syncytial layer and multicompartmental embryonic barriers. Development of a non-invasive tool for manipulating embryos would not only benefit the field of developmental biology, but also impact both present and future advancements in cryopreservation, medical research and the aquaculture industry [11,12].

Several reports have documented the application of laser pulses for ablating the zebrafish. Conventional laser systems that have been used for ablation include the Q-switched neodymium:yttrium-aluminum garnet laser and the Micropoint nitrogen gas laser, both operating at a pulse duration of nanoseconds (ns) [13-16]. Using ns laser pulses with wavelengths in the ultraviolet (UV) and visible spectra, ablation of larval melanocytes [15], pigments cells [14], sensory cells [16] and the optic tectum [13] have been demonstrated. While the ablation performed by these laser systems has been invaluable to the study of zebrafish, the targeting of deep tissue structures remains a challenge. This difficulty is inherent to UV wavelengths since biological tissues are strongly absorbing, and UV photons experience strong scattering in deep tissue studies [17]. Furthermore, localized ablation using conventional laser systems is challenging, as neighbouring cells within the focused light cone are disrupted, as opposed to single cell ablation at the laser focus. Near- to mid-infrared (NIR-MIR) optical wavelengths are ideal for deep tissue ablation, where scattering and radiation loss are significantly reduced [17]. Using NIR to MIR photons, ablation is induced only at the laser focus where non-linear multiphoton absorption occurs. Consequently, tissue disruption is localized to the laser focal plane of the light cone, rendering cells above and below the focus unaffected. Pulse duration is also an important parameter that must be carefully chosen in laser-tissue interaction studies. Ablation using ns laser pulses requires higher threshold energies than ablation performed using fs laser pulses [17-19]. Consequently, lower threshold energies result in lower energy conversion into adverse mechanical and thermal side effects [20].

Recently, the application of fs laser pulses has shown promising results as a tool for manipulating biological material. Since fs laser pulses can be focused to a sub-micron to near diffraction limited focal spot, fluorescently labeled cellular and sub-cellular components can be precisely targeted without disrupting material above or below the laser focal spot. Thus, key features within structurally complex organisms can be easily targeted in a contact-free manner. Fs laser pulses have been successfully used in the cell isolation and nanodissection of focal adhesions in mammalian cells [21], the nanoprocessing of plastids in plants [22], the ablation of sub-cellular structures in the cytoskeleton [23], the ablation of human metaphase chromosomes [24], and the opto-injection of non-reducing disaccharides for biopreservation [25]. Each of these applications has been achieved with minimal or no effect on the viability of the biological system studied. Recently, in a report by our group [26], we demonstrated the exogenous delivery of Streptavidin-conjugated quantum dots and a reporter gene (EGFP) into the embryonic cells of early stage zebrafish embryos using fs laser pulses. Streptavidin-conjugated quantum dots were introduced into the blastomeres of early stage dechorionated embryos and were found to freely diffuse into and throughout the blastomeres [26]. After laser transfection of a plasmid into early stage dechorionated embryos, expression was observed at 24 hours post-fertilization along the yolk-extension, notochord, floor plate, somites and tail cells [26]. However, it remains to be determined if the application of fs laser pulses induces long-term effects on the progeny of mammalian cells or abnormal long-term development of early stage embryos. For proper quantification of long-term effects, developmental changes induced by the laser should be studied *in vivo*, unlike the aforementioned studies [21-25]. Early stage zebrafish embryos represent an ideal model system for performing such work. Adverse effects on development must be recognized before the integration of this tool into the life sciences is realized. To our knowledge, this is the first detailed study documenting the effects of fs laser pulses on both the short- and long-term development of zebrafish embryos.

**Figure 1:**
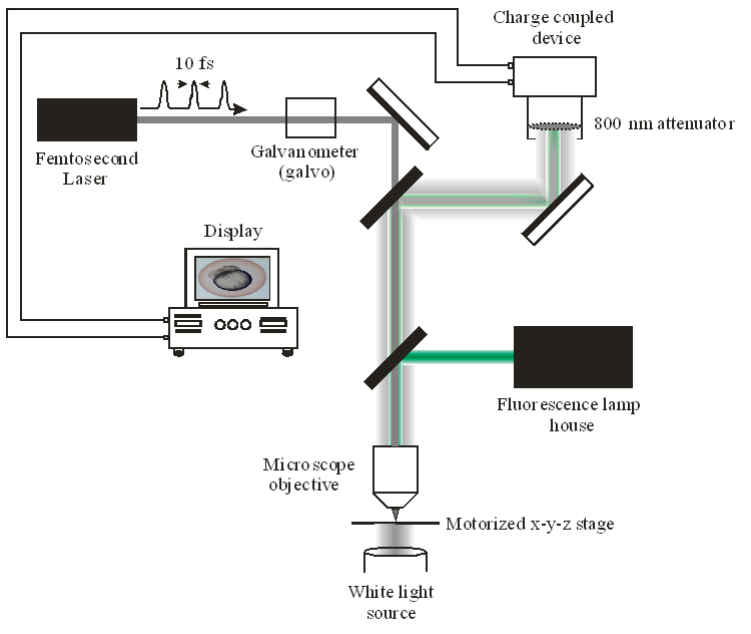
The optical setup used in the laser manipulation of early to mid cleavage stage (2 to 4/8 cell) zebrafish embryos. Sub-10 fs laser pulses were generated from a Kerr lens modelocked titanium sapphire laser oscillator. The centre wavelength of the laser pulses was at 800 nm, with a pulse repetition rate of 80 MHz. Fs laser pulses were focused by a 1.0 NA water immersion microscope objective to a focal spot of ~800 nm. A galvanometer was used to select the beam dwell time irradiating the embryos. The embryos were placed on a motorized *x*-*y*-*z *stage, with an in plane translation speed of 1 mm/sec and a *z*-focus step resolution of 50 nm. White light illuminated the samples in the inverted position. For fluorescence assessment, fluorescence excitation was collinearly coupled through the objective onto the sample. A charge-coupled device (CCD) was used to observe the laser-manipulation of the embryos. The CCD was interfaced with a computer, allowing for video capture and image analysis.

In this article, we investigated fs laser pulse manipulation of early to mid cleavage stage (2-cell to 4/8-cell) zebrafish embryos. Using a range of average laser powers and beam dwell times, surgical-ablation of individual blastomere cells was performed, Figure 1. Based on visual assessment of the ablated blastomeres, optimal laser parameters were determined. Optimal parameters were defined as the minimum average laser power and beam dwell time required for surgical-ablation. To determine whether these laser parameters could be used for transient pore formation and the intracellular introduction of exogenous material, embryos were laser ablated near the blastomere-yolk interface in the presence of a fluorescent probe. The embryos were then examined for fluorescence, Figure 1. To further refine the laser parameters, transient pores were formed in the blastomeres using varying beam dwell times to visually monitor changes in the intracellular fluorescence intensity. Using the refined parameters, both early cleavage stage (2-cell) laser manipulated and control embryos were reared until 2 days post-fertilization (dpf) to determine short-term survival. Embryo survival was assessed by comparing developmental morphology and hatching rates using light microscopy (LM) and scanning electron microscopy (SEM). Since adverse effects induced by the laser may not be apparent until later developmental stages, we also compared the developmental morphology of laser manipulated and control embryos that were reared until 7 dpf. SEM mosaics of entire larvae and dorso-lateral views of the olfactory pit, ear, hind- and midbrain were examined to determine long-term morphological survival. The work presented in this paper provides the basis for future studies of fs laser interactions with multicellular biological systems.

### Note on figures

The fluorescence, LM and SEM images included in this article represent a subset of the experimental specimens that clearly demonstrate the observations discussed below. All observations and conclusions were based on the entire sample group.

### Terminology defined

Throughout the rest of the text we define 'laser-manipulated larva' as an embryo laser treated at the early to mid cleavage stage (2-cell to 4/8-cell), and subsequently allowed to hatch into a larva and mature to the indicated developmental endpoint (2 or 7 dpf).

## Results

### Surgical-ablation of individual blastomeres at the early to mid cleavage stage

**Figure 2:**
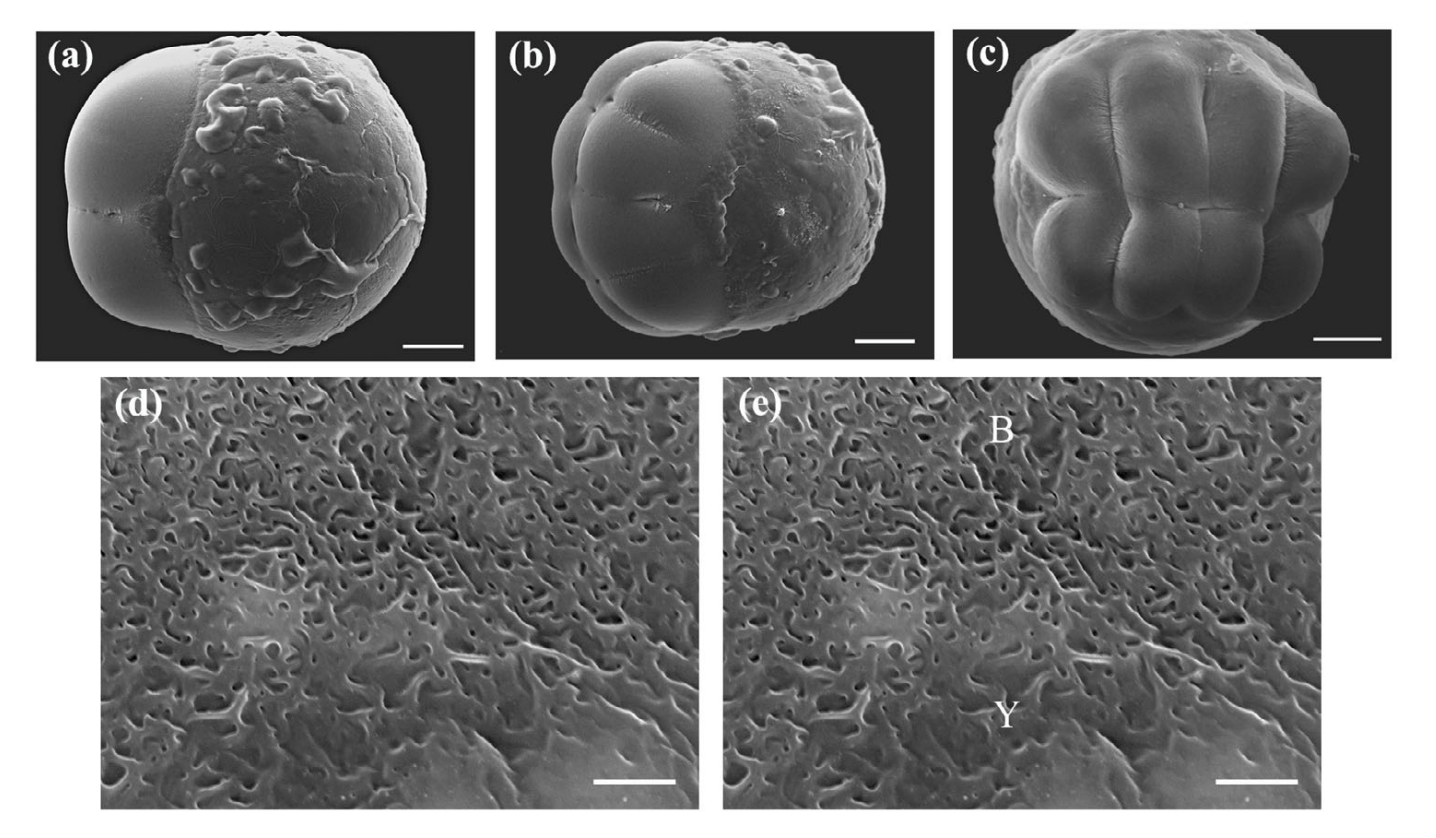
Face, animal-vegetal axis rotated and top views of (a) 2-cell stage embryo and (b, c) 8-cell stage embryo. (d, e) High magnification SEM images of the blastomere-yolk interface of an 8-cell stage embryo. Scale bar for (a, b, c) is 100 μm and (d, e) 5 μm.

To determine the optimal laser parameters for delivery of an exogenous fluoresecent probe, individual dechorionated blastomeres at the early to mid cleavage stage (2-cell to 4/8-cell) were laser ablated (n = 30) using average laser powers and beam dwell times of 25 to 50 mW and 5 to 500 ms respectively. A galvanometer (galvo) was used to control the beam dwell time, with a shortest gating window time of 5 ms. For each average laser power and beam dwell time used, blastomeres were surgically-ablated near the blastomere-yolk interface by a single pulsing event of the galvo. On average, 4 to 5 ablation spots were made in each cell, with a maximum of 2 to 4 laser-treated blastomeres per embryo. Figures 2(a, b,c) depict control SEM images of face, animal-vegetal axis rotated and top views of dechorionated embryos at 2- and 8-cell stage. High magnification SEM images of the blastomere-yolk interface of a control 8-cell stage embryo are presented in Figures 2(d,e).

Laser-induced surgical-ablation was assessed by visual inspection. Specifically, we looked for the formation of cavitations (gas bubbles formed from the high laser intensity at the focal plane) and for residual scarring of the blastomeres post-manipulation. Optimal laser parameters for surgical-ablation were defined as both the minimum average laser power and beam dwell time that visually produced ablated blastomeres. At the lowest average laser power of 25 mW, the region near the blastomere-yolk interface could not be surgically ablated. This result was independent of the beam dwell time. Increasing the average laser power to 30 mW, ablation was observed with high reproducibility for beam dwell times in excess of 500 ms. However, at lower beam dwell times of 50 to 200 ms, surgical-ablation near the interface occurred only occasionally. Increasing the average laser power to 40 to 45 mW, surgical-ablation near the blastomere-yolk region occurred independently of the chosen beam dwell time. We observed the presence of cavitation bubbles for the entire range of beam dwell times, with the bubbles decaying within a few seconds for times ≤ 100 ms. Beyond 45 mW, ablation persisted over the entire beam dwell range, however larger cavitation bubbles were observed along with increased blastomere-surface damage over a larger area. Based on our previous definition of optimal parameters, average laser powers of 40 to 45 mW with beam dwell times ≤ 100 ms were ideal for embryo surgical-ablation. Using these parameters, precise ablation of the blastomere-yolk region could be achieved. Table 1 presents a summary of the results.

**Table 1:** Laser surgical-ablation of individual blastomere cells – Summary

Average laser power	Beam dwell time	Observations
25 mW	5 to 500 ms	Region near the blastomere-yolk interface could not be laser ablated. This result was independent of the chosen beam dwell time.
30 mW	50 to 200 ms	Region near the blastomere-yolk interface was occasionally laser ablated.
	> 500 ms	Laser ablation near the blastomere-yolk region occurred with high reproducibility.
40–45 mW	5 to 500 ms	Surgical-ablation near the blastomere-yolk region occurred for the entire beam dwell range. Cavitation bubbles were short lived for beam dwell times ≤ 100 ms. Above 100 ms, cavitation bubbles were large, with increased blastomere-surface damage.
> 45 mW	5 to 500 ms	Surgical-ablation persisted for the entire beam dwell range. Laser induced cavitation bubbles were large. A large area of blastomere-surface damage was observed.

### Transient pore formation and exogenous FITC delivery

Delivery of exogenous foreign material requires pore formation that exposes the extracellular space to the intracellular environment. Transient pores are ideal, as the pore closes shortly after creation, thereby maintaining homeostasis. The transient pores provide a transport pathway for delivering impermeable molecules into the blastomeres of the developing embryo. Using the previously determined optimal laser parameters, we addressed whether these laser conditions could be used to induce transient pore formation in the region near the blastomere-yolk interface. Early cleavage stage (2-cell) dechorionated embryos (n = 10) were laser ablated with an average laser power of 45 mW and a beam dwell time of 100, 50 or 20 ms. These three beam dwell times were chosen to examine changes in the signal intensity of the exogenously delivered molecule. In each case the galvo was pulsed 1 to 3 times per pore (the time between each galvo pulsing event was < 20 seconds) to further refine the laser conditions for transient pore formation. At the time of laser pulse induced surgical-ablation, each embryo was at the 2-cell stage. A total of 3 transient pores per blastomere were created in each of the two cells (6 pores created per embryo). Since a batch of embryos was laser ablated sequentially, it was expected that the embryos ablated earlier in the sequence would have developed slightly ahead of the later embryos. The maximum lag time between the embryos was observed to be approximately one cleavage, corresponding to a developmental time of 12 to 15 minutes [27]. Developmental delays between embryos were not expected to affect the results presented below.

**Figure 3:**
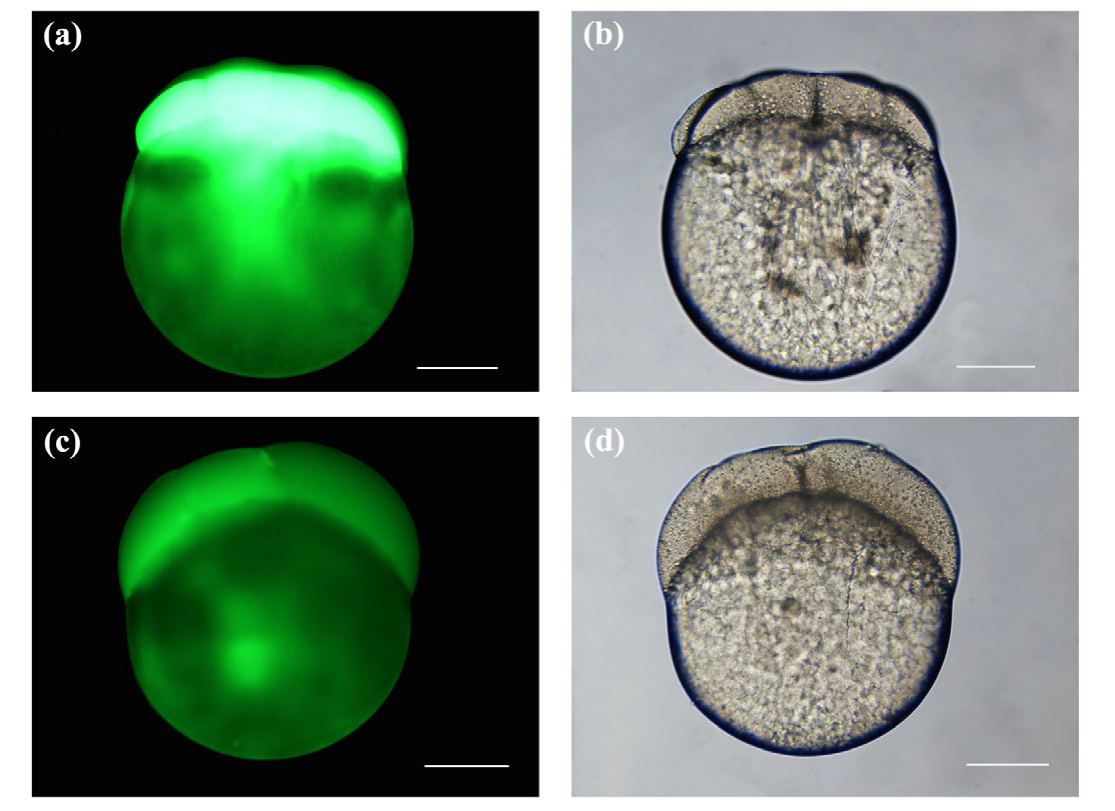
Laser-manipulated embryos porated for exogenous FITC delivery using an average laser power of 45 mW. (a, b) Fluorescence and brightfield image of a post-laser manipulated 8-cell stage embryo. A total of 6 pores were made in 2 cells, 3 pores per blastomere, using a beam dwell time of 100 ms and 3 pulsing events of the galvo. (c, d) Fluorescence and brightfield image of a post-laser manipulated 4-cell stage embryo. Same number of pores were made in the blastomeres as indicated in (a, b). Beam dwell time was set to 100 ms and the galvo was pulsed once. Scale bars for (a, b, c, d) represent 200 μm.

Figures 3(a, b) depict post-laser ablated embryos at 8-cell stage porated using an average laser power of 45 mW and a beam dwell time of 100 ms. Each of the 6 pores was a result of 3 pulsing events of the galvo at 100 ms. As shown in Figure 3(a), strong fluorescence was observed in the blastomeres of the developing zebrafish embryo, indicating that transient pores were formed and that exogenous FITC was delivered into the blastomere cells. The embryo remains intact post-manipulation, as can be seen in the brightfield image of Figure 3(b) by the lack of cytoplasmic leakage into the extracellular environment. Figures 3(c,d) represent post-laser manipulated embryos at 4-cell stage. Here, blastomeres were surgically-ablated at the same average laser power as in Figures 3(a,b), however, each pore was formed by a single pulsing event of the galvo at 100 ms. (Data for pores created using 2 pulsing events of the galvo are not shown). Clearly, the fluorescence signal in Figure 3(c) is considerably less intense than that in Figure 3(a), with the leftmost blastomere in Figure 3(c) exhibiting slightly higher fluorescence intensity. Lower fluorescence in Figure 3(c) likely reflects the kinetics of the transient pore, where the pore seals before a significant accumulation of intracellular FITC. It should be noted that in Figures 3(a) and 3(c), weak fluorescence was also observed in the yolk. This autofluorescence does not represent fluorescence from FITC fluorophores since it is our hypothesis that the yolk platelets are impermeable to FITC.

**Figure 4:**
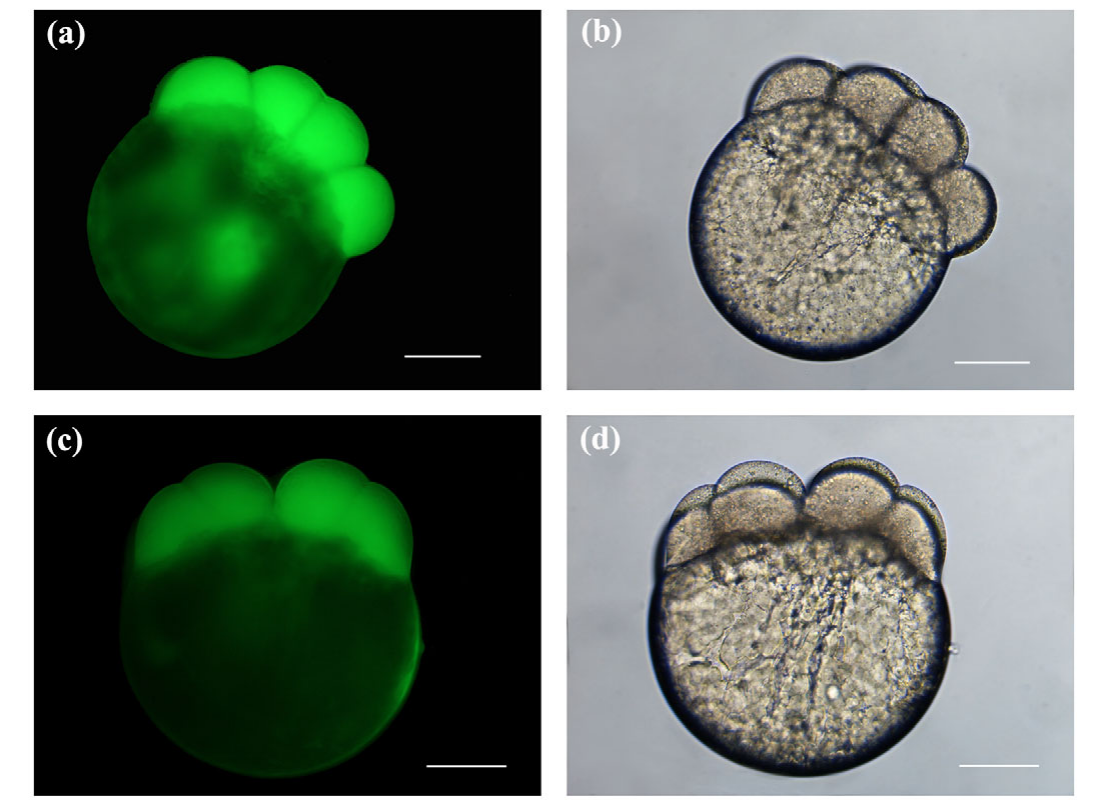
Fluorescence and brightfield images of post-laser manipulated 8-cell stage embryos. Embryos were porated for exogenous FITC delivery using an average laser power of 45 mW. The beam dwell time was set to 50 ms with three pulsing events of the galvo for (a, b) and two for (c, d). Three pores were made in each of 2 blastomere cells, yielding a total of six pores for the entire embryo. Scale bars for (a, b, c, d) represent 200 μm.

Figures 4(a, b, c, d) depict fluorescence and brightfield images of post-laser ablated embryos at 8-cell stage. Embryos were ablated using an average laser power of 45 mW and a beam dwell time of 50 ms. In Figures 4(a,b), the galvo was pulsed three times per pore, and in Figures 4(c,d) twice per pore. Comparing Figure 4(a) with Figure 4(c), the fluorescence signal was noticeably stronger in Figure 4(a). Both images display fluorescence in all blastomeres, since transient pores were formed in both cells of the early 2-cell stage embryos. The brightfield images of Figures 4(b) and 4(d), showing no observable cytoplasmic leakage, confirm that the embryos were not structurally compromised post-manipulation.

**Figure 5:**
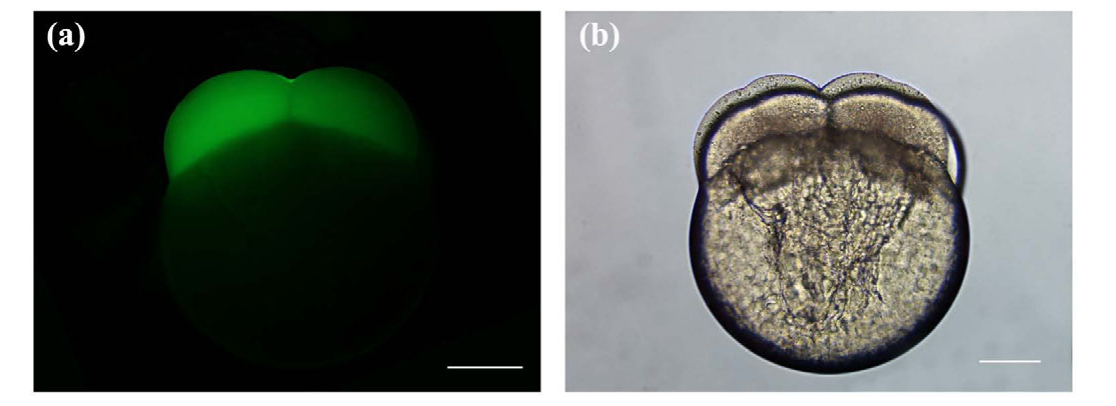
Fluorescence and brightfield image of a post-laser manipulated embryo at 8-cell stage. (a, b) Embryo was porated for exogenous delivery using an average laser power of 45 mW with a beam dwell time set to 20 ms and a galvo pulse rate of three. Three pores were made in each of 2 blastomere cells, yielding a total of six pores for the entire embryo. Scale bars for (a, b) represent 200 μm.

Using an average laser power of 45 mW and a beam dwell time of 20 ms, Figures 5(a, b) show a post-laser porated 8-cell stage embryo. Each pore resulted from three pulsing events of the galvo at 20 ms. The fluorescence intensity in Figure 5(a) was relatively weak, however FITC was observed in all blastomeres.

**Figure 6:**
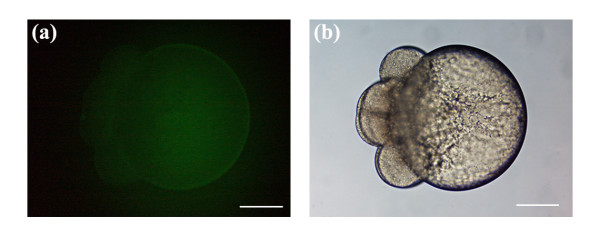
(a, b) Fluorescence and brighfield image of an 8-cell stage embryo that was not laser-manipulated, but was bathed in a concentration of 0.014 to 0.018 mg/ml FITC-tank water for 10 to 15 min. The embryo was rinsed several times in fresh tank water and subsequently imaged with an imaging area and exposure time consistent with Figures 3, 4 and 5. No FITC dye was observed in the blastomere cells. (a) Weak autofluorescence was observed in the yolk. Scale bars for (a, b) represent 200 μm.

Ablation and pore formation are necessary requirements for the exogenous delivery of FITC into the embryonic cells. FITC is impermeable to the blastomeres, and the delivery of the dye into the cells, as observed in Figures 3, 4 and 5, must have occurred through the transient pores. This is in agreement with our previous work [26] and is evidenced in Figure 6. The 8-cell stage dechorionated embryo was bathed in FITC and subsequently washed without being laser-manipulated. Figure 6(a) depicts the fluorescence image of the embryo after exposure to FITC with no irradiation (Note: The embryo was exposed to FITC and imaged using the same exposure parameters as Figures 3, 4 and 5). No dye was observed in the blastomere cells, confirming that exogenous FITC delivery into the embryonic cells must occur through laser-induced transient pores. The weak fluorescence that is observed in the yolk is autofluorescence.

**Figure 7:**
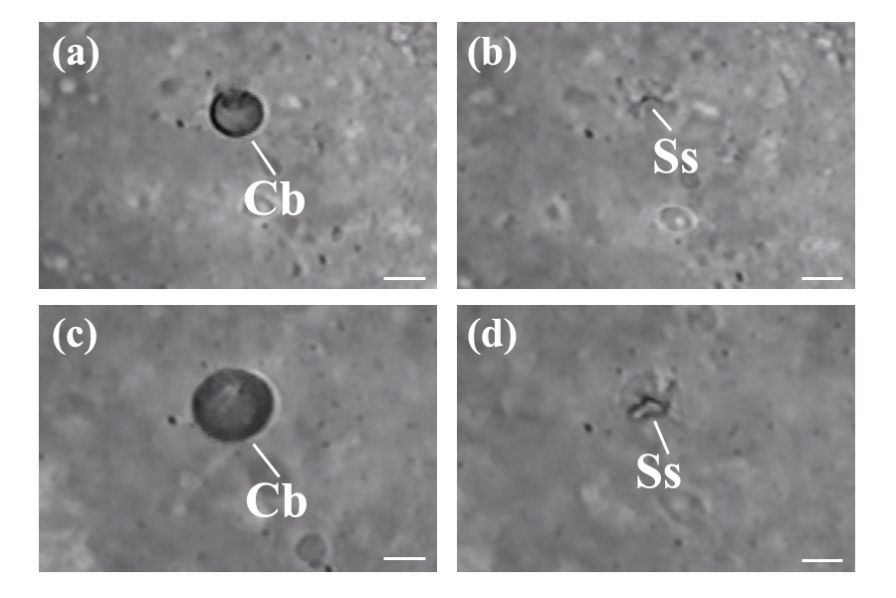
(a, b, c, d) Depicts cavitation bubbles (Cb) and residual surface scarring (Ss) after the applied fs laser pulses. (a) Cavitation bubble (Cb) was produced using an average laser power of 45 mW with a beam dwell time of 100 ms and a single pulsing event of the galvo. The diameter of the cavitation bubble was measured to be ~5 μm. (b) After pulsing the galvo a total of 3 times, surface scarring (Ss) of the blastomere was observed. The spatial extent of tissue scarring was measured to be ~5 μm. (c) Depicts the cavitation bubble (Cb) created using an average laser power of 45 mW with a beam dwell time of 500 ms and a single pulsing event of the galvo. Diameter of the cavitation bubble was measured to be ~10 μm. (d) After pulsing the galvo a total of 2 times, surface scarring (Ss) of the blastomere was observed. The spatial extent of tissue scarring was measured to be ~10 μm. (a, b, c, d) Still images were extracted from additional file 1. Scale bars for (a, b, c, d) represent 5 μm.

Figure 7 depicts the cavitation bubbles and tissue scarring after fs laser ablation near the blastomere-yolk interface of a 2-cell stage chorionated embryo. In Figure 7(a), the cavitation bubble (Cb) was formed using an average laser power of 45 mW and a beam dwell time of 100 ms (the galvo was pulsed once). The cavitation was measured to be ~5 μm in diameter. Figure 7(b) shows surface scarring (Ss) of the blastomere after ablation using the same average laser power and beam dwell time, but with the galvo pulsed a total of 3 times. When the beam dwell time was increased to 500 ms, Figure 7(c), the cavitation bubble increased in diameter to ~10 μm (the galvo was pulsed once). Figure 7(d) depicts the surface scarring (Ss) after pulsing the galvo a total of 2 times. Comparing the scarring observed in Figure 7(b) with Figure 7(d), the scarring length was measured to be ~10 μm for Figure 7(d) and ~5 μm for Figure 7(b). Live video of the ablation is provided in Additional file 1. The images presented in Figure 7 were still images extracted from the video.

The previous findings demonstrated that transient pores could be formed in the blastomere cells of early stage embryos using the optimal average laser power (45 mW) previously defined. The fluorescence images in Figures 3, 4, 5 and 6 were obtained using a constant imaging area and an exposure time of 1.5 seconds. Comparing Figures 3, 4 and 5, a general trend towards decreasing blastomere fluorescence intensity was observed as both the beam dwell time and galvo pulsing number decreased. This observation was based on visual inspection of the fluorescence intensity seen in Figures 3, 4 and 5, and represents a qualitative assessment of the fluorescence. Visual observation of blastomere fluorescence was sufficient to obtain informative results in this study as well as to identify the trend in fluorescence intensity as a function of beam dwell time and galvo pulse rate. However, to provide a more accurate analysis of the blastomere fluorescence, and to confirm the qualitative conclusions, we measured the mean fluorescence intensity per unit area of the embryos presented in Figures 3, 4, 5 and 6. The respective mean intensities per unit area were 53.9 (100 ms; 3 pulsings of the galvo), 16.9 (100 ms; 1 pulsing of the galvo), 29.7 (50 ms; 3 pulsings of the galvo), 15.4 (50 ms; 2 pulsing of the galvo), 15.0 (20 ms; 3 pulsing of the galvo) and 4.4 (autofluorescence; non-laser-manipulated) for Figures 3, 4, 5 and 6. Since FITC was delivered directly into the cells, the above measurements represent the mean fluorescence intensity per unit area from the blastomeres only. From the above values, we notice that the blastomere fluorescence intensity decreases with decreasing beam dwell time and galvo pulse number. These quantitative measurements confirm our qualitative assessment. The mean fluorescence intensity per unit area for Figure 6 was considerably lower than the values for Figures 3, 4 and 5, confirming that transient pore formation was required for exogenous delivery of FITC. It should be noted that both the quantitative and qualitative analysis of the blastomere fluorescence showed that the fluorescence intensity for 50 ms and 3 pulsing events of the galvo (29.7 mean intensity/area) was higher than that for 100 ms and 1 pulsing event of the galvo (16.9 mean intensity/area). This was expected, since the total beam dwell time was longer, 150 ms (50 ms × 3) vs. 100 ms (100 ms × 1), for 50 ms and 3 pulsing events of the galvo (see discussion).

In disciplines like cryopreservation and developmental biology, achieving the highest intracellular concentration of a desired exogenous compound (i.e. cryoprotective molecule or gene of known function) is important. Based on the above findings, an average laser power of 45 mW with a beam dwell time of 100 ms and a galvo pulse number of 3 represents the ideal choice for poration and exogenous delivery. It remains to be determined whether these laser parameters induce short- and long-term effects on the development of the zebrafish embryo.

### Short-term survival assessment of laser-porated embryos at early to mid cleavage stage

To determine whether the laser induced short-term effects on embryo development, both control (n = 30) and laser porated (n = 30) 2/4-cell stage embryos were reared at 27 ± 1°C until 2 dpf. (Note: all embryos were laser treated using an average laser power of 45 mW with a beam dwell time of 100 ms and a galvo pulse number of 3. A total of 3 transient pores per blastomere were created with a maximum of two cells laser manipulated). The media surrounding the embryos was replaced with fresh tank water 1 to 2 times per day to avoid bacterial or fungal growth and the accumulation of protozoans that feed on developing embryos. (Dyes such as methylene blue are known to reduce fungal and bacterial growth, and can be added to the solution. Methylene blue was not used in our experiments as the dye both permeates and stains the chorion, making the developmental stage of the embryo difficult to assess). In order to obtain accurate survival assessments all embryos remained chorionated and were not exposed to the fluorescent probe since remnants of the proteolytic enzyme used for dechorionation, as well as the cytotoxicity induced by the fluorescent probe can affect short- and long-term survival. Using the optimal laser parameters for poration and exogenous delivery, laser pulses were focused beyond the chorion for pore formation near the blastomere-yolk interface. Focusing the pulses in this manner did not compromise the chorion. It should be noted that this method of targeting beyond the chorion is unique to this reported tool, and cannot be accomplished with other techniques. During the course of the experiment we did not observe increased difficulty in porating chorionated embryos, indicating that energy loss through the chorion was minimal.

Embryo survival was determined by comparing the hatching rates and developmental morphologies of laser-manipulated larvae against control larvae. Using LM, the body plans of control and laser-manipulated larvae were examined. In this study, any laser-manipulated larvae with significant developmental differences and hatching rates relative to the control were considered morphologically compromised. We also examined the larvae for asymmetric yolk sacs and abnormal dorsal curvature or development of the body axis. Based on the above analysis, larvae were categorized as either being viable or non-viable. From this developmental assessment, the short-term survival of laser-manipulated larvae was found to be 93% (28/30). Viable larvae showed no differences in hatching rates as compared to the controls, and developmental features looked very similar to those of control larvae.

**Figure 8:**
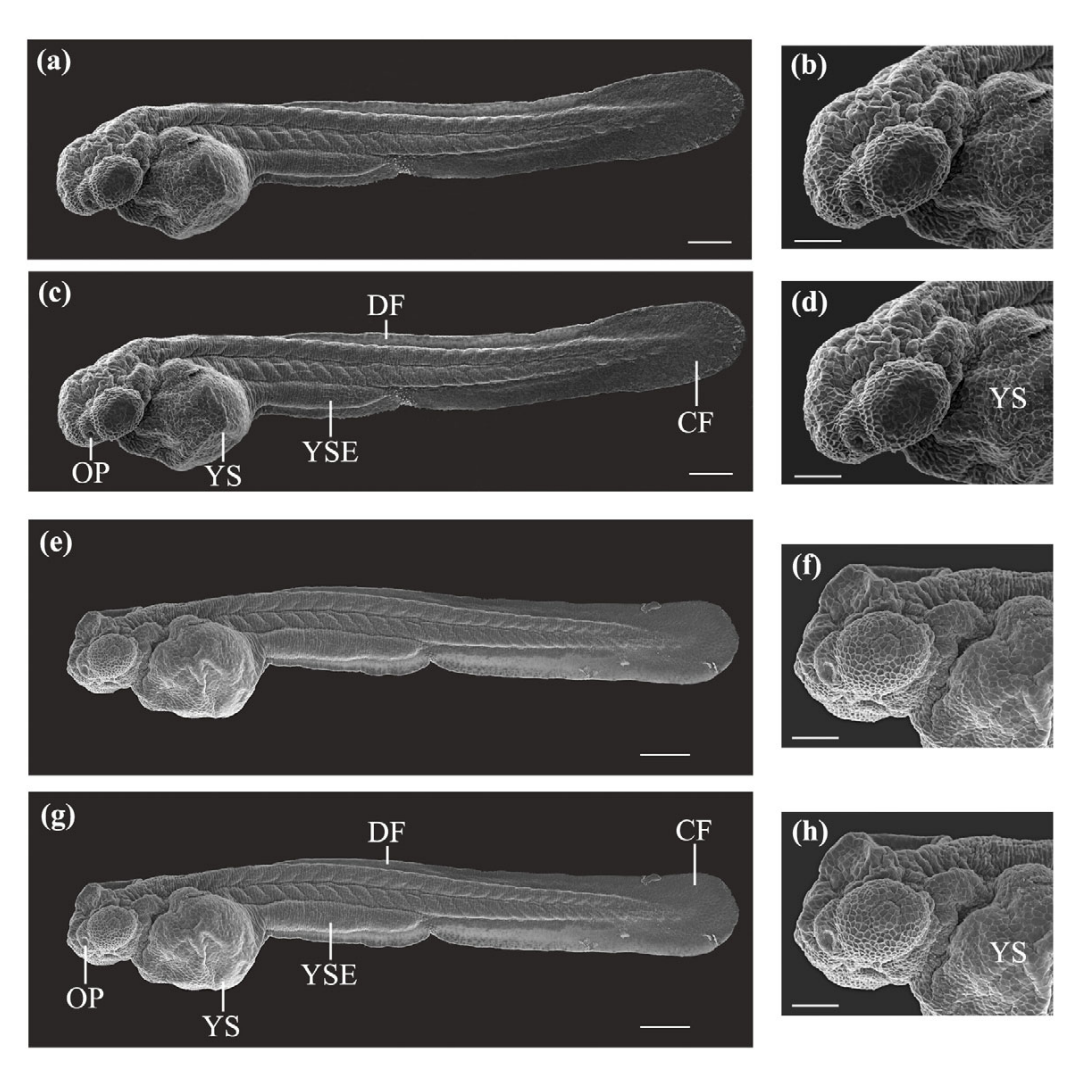
SEM images of a laser-manipulated and a control larva reared to 2 dpf. (a, b) Whole body and antero-lateral views of a laser-manipulated larva at 2 dpf. (c, d) Same larva as in (a, b). Key developmental features that are illustrated are the yolk sac (YS), yolk sac extension (YSE), dorsal fin (DF), caudal fin (CF) and the olfactory pit (OP). (e, f) Whole body and antero-lateral views of a control larva at 2 dpf. (g, h) Same larva as in (e, f). The same developmental features observed in (c, d) were also seen in (g, h). Scale bars for (a, c, e, g) represent 200 μm and for (b, d, f, h) 100 μm.

While visual inspection of larvae using LM provided a general assessment of development, sub-micrometer developmental features could not be resolved using optical microscopy. SEM provided an ideal tool for high resolution imaging, allowing structures below 0.5 μm to be resolved. Figure 8 depicts SEM images (n = 8 control; n = 10 laser) of a laser-manipulated larva (Figures 8(a,b,c,d)) and a control larva (Figures 8(e,f,g,h)) at 2 dpf. Figures 8(a,c,e,g) show lateral views of whole larvae, while Figures 8(b,d,f,h) represent magnified antero-lateral views. The control larva and the laser-manipulated larva were very similar in terms of body plan. In Figures 8(a,c) and 8(e,g), both larvae have their heads curved over the yolk sac (YS) pointing ventrally. Key developmental features observed in the control, Figures 8(e,g), were also seen in the laser-manipulated larva, Figures 8(a,c), and include the yolk sac (YS), yolk sac extension (YSE, also known as the gut), dorsal fin (DF), caudal fin (CF) and the olfactory pit (OP). The 93% survival rate and the results presented in Figure 8 indicate that the laser does not induce any significant short-term effects on the development of the zebrafish embryo.

### Long-term effects of laser poration on early to mid cleavage stage embryo development

**Figure 9:**
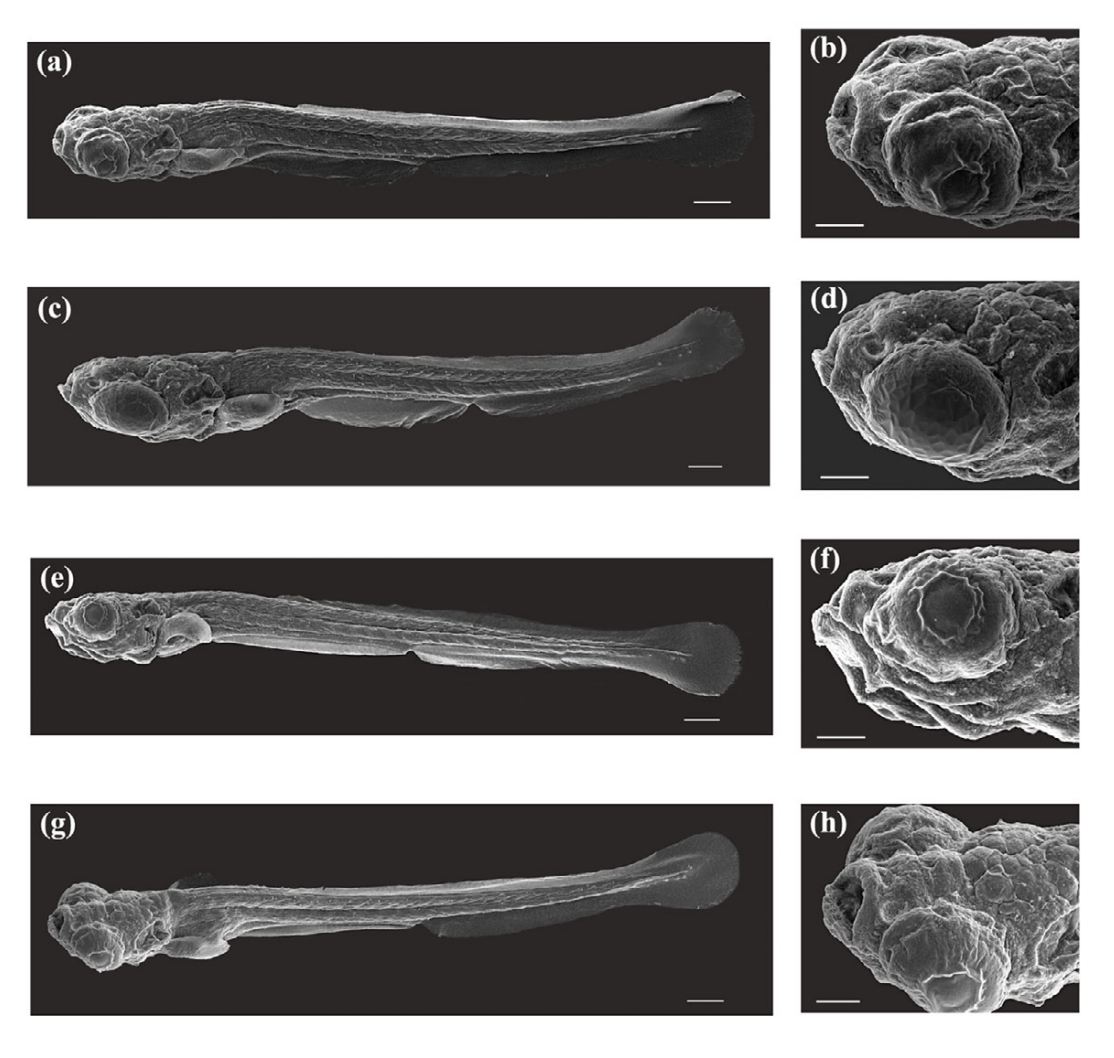
SEM images of laser-manipulated and control larvae reared to 7 dpf. (a, b, c, d) Dorso-lateral whole body views and antero-dorsal views of laser-manipulated larvae at 7 dpf. (e, f, g, h) Dorso-lateral whole body views and antero-dorsal views of control larvae at 7 dpf. Scale bars for (a, c, e, g) represent 200 μm and for (b, d, f, h) 100 μm.

**Figure 10:**
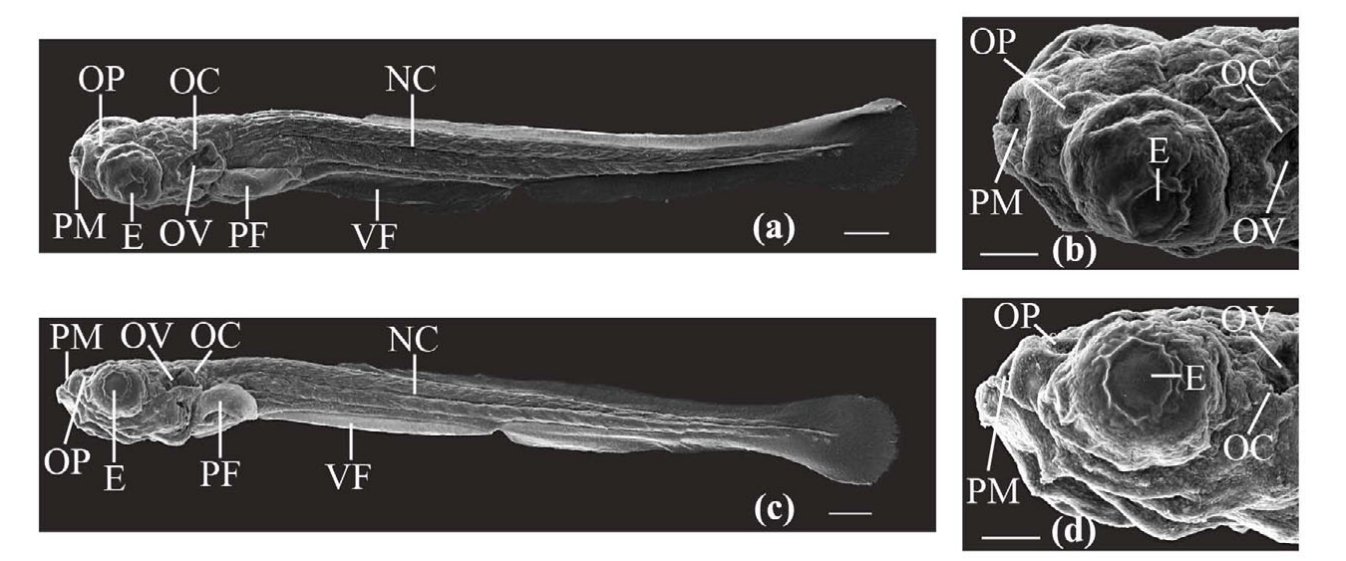
Key developmental features in a laser-manipulated and a control larva reared to 7 dpf. (a) SEM whole body image of a laser-manipulated larva at 7 dpf. Developmental features indicated are the ventral fin (VF), notochord (NC), pectoral fin (PF), otic capsule (OC), otic vesicle (OV), eye (E; cornea), olfactory pit (OP) and the protruding mouth (PM). (b) Magnified antero-dorsal view of the protruding mouth (PM), olfactory pit (OP), eye (E; cornea), otic capsule (OC) and otic vesicle (OV) of the larva in (a). (c) SEM whole body image of a control larva at 7 dpf. Similar developmental features observed in (a) were also seen in (c). (d) Magnified antero-lateral view of the same larva in (c). Developmental features illustrated in (b) are also shown in (d). Scale bars for (a, c) represent 200 μm and for (b, d) 100 μm.

Since the laser's effect on embryo development may not be apparent until later developmental stages, control (n = 12) and laser-manipulated larvae (n = 10) at 2/4-cell stage were reared at 27 ± 1°C until 7 dpf and visually inspected for abnormal growth. (Note: all embryos were laser treated using an average laser power of 45 mW with a beam dwell time of 100 ms and a galvo pulse number of 3. A total of 3 transient pores per blastomere were created with a maximum of two cells laser manipulated). Larvae were examined using LM, and developmental features of laser-manipulated larvae such as the protruding mouth, olfactory pit, pectoral fin, otic capsule and otic vesicle were compared against those of the control larvae. We observed no differences in developmental morphology between the samples. Figure 9 depicts SEM images (n = 9 control; n = 10 laser) of dorso-lateral, lateral and dorsal views of laser-manipulated (Figures 9(a,b,c,d)) and control (Figures 9(e,f,g,h)) larvae developed to 7 dpf. Figures 9(a,c,e,g) represent lateral and dorso-lateral views of whole larvae, while Figures 9(b,d,f,h) are magnified lateral and dorsal views of the head. Whole laser-manipulated larvae looked developmentally similar to control larvae. This is evident in Figure 10, which depicts developmental features in a control larva (Figures 10(c,d)) that were also observed in a laser-manipulated larva (Figures 10(a,b)). The observed developmental characteristics were the ventral fin (VF), notochord (NC), pectoral fin (PF), otic capsule (OC), otic vesicle (OV), eye (E; cornea), olfactory pit (OP) and the protruding mouth (PM). Magnified views of the protruding mouth (PM), olfactory pit (OP), eye (E; cornea), otic capsule (OC) and otic vesicle (OV) of both larvae are shown in Figures 10(b) and 10(d).

**Figure 11:**
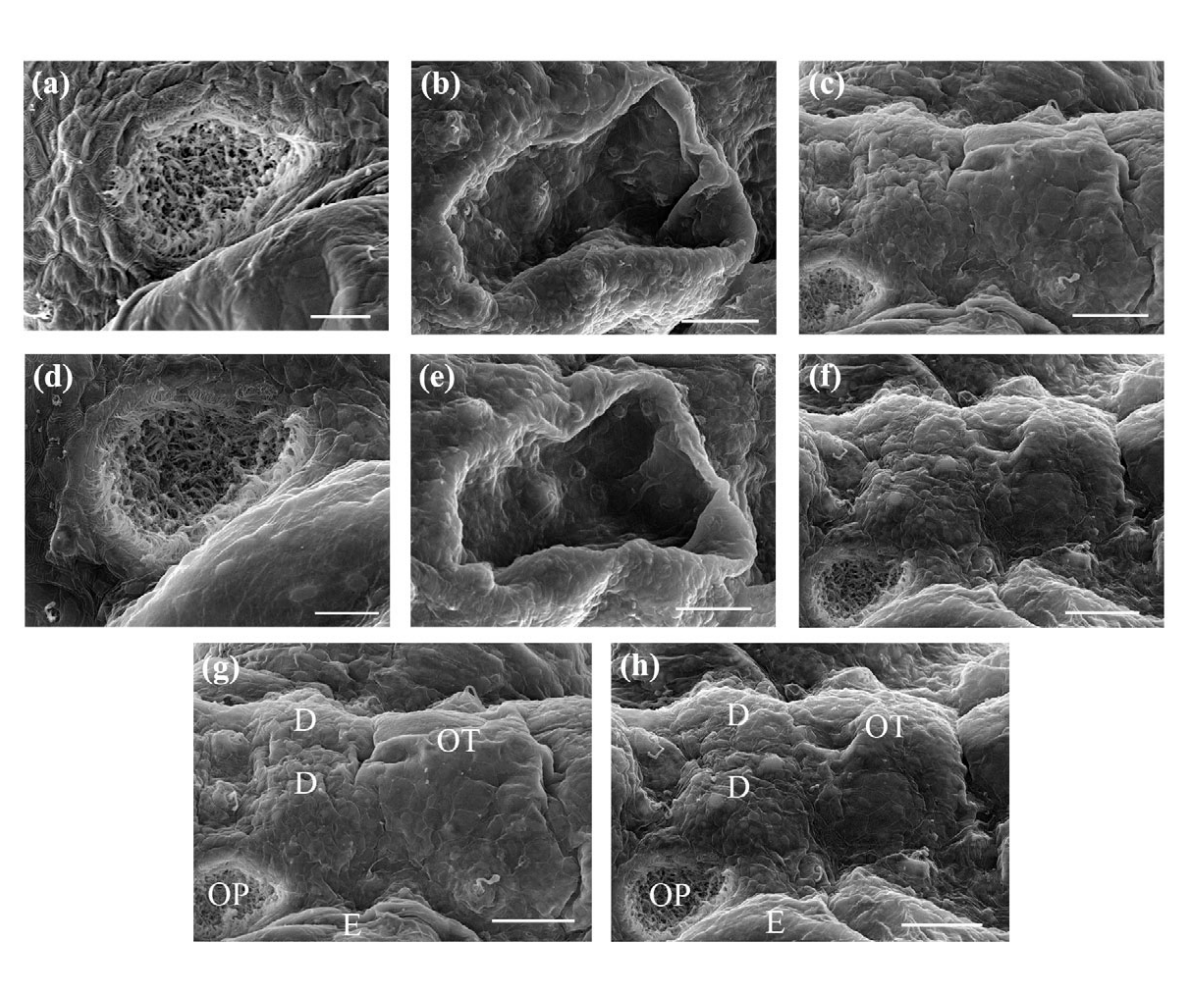
Magnified top and lateral SEM images of key developmental features in larvae reared to 7 dpf. (a, b, c) Depict the olfactory pit, ear, posterior forebrain and dorsal midbrain in laser-manipulated larvae. (d, e, f) Depict the olfactory pit, ear, posterior forebrain and dorsal midbrain in control larvae. (g, h) Illustrated comparison of dorso-lateral views of the posterior forebrain and dorsal midbrain in (g) a laser-manipulated and (h) a control larva at 7 dpf. Location of the eye (E), diencephalon (D) and optic tectum (OT) are shown respectively. Scale bars for (a, d) represent 20 μm and for (b, c, e, f, g, h) 50 μm. Orientation: anterior (left); posterior (right).

Further evidence of normal development in laser-manipulated larvae is shown in the high-magnification images presented in Figure 11. The SEM images represent magnified dorsal and lateral views of both control (Figures 11(d,e,f,h)) and laser-manipulated larvae (Figures 11(a,b,c,g)) at 7 dpf. For laser-manipulated larvae, the olfactory pit (OP), otic capsule (OC), otic vesicle (OV), posterior forebrain and dorsal midbrain are illustrated in Figures 11(a,b,c). Figures 11(d,e,f) show the same developmental features in control larvae. A comparison between dorso-lateral views of a laser-manipulated larva (Figure 11(g)) and a control larva (Figure 11(h)) is presented. Key developmental features that have been emphasized are the olfactory pit (OP), diencephalon (D) and optic tectum (OT). The telencephalon is anterior to the diencephalon and the cerebellum is posterior to the optic tectum. The striking similarities between developmental features in the laser-manipulated and control larvae indicate that the laser does not adversely affect long-term zebrafish development. Additional file 2 depicts normal development of a laser manipulated 2/4-cell stage embryo that was reared to 5 dpf.

## Discussion

### Laser ablation and poration

When fs laser pulses are focused to a sub-micron spot, non-linear multiphoton absorption of laser light at the focus results in the electronic excitation of valence electrons to the conduction band. If the peak intensity at the focus is large, on the order of 10^12 ^to10^13 ^W/cm^2^, ionization of electrons occurs. These electrons, termed "seed electrons" provide the initial carriers for laser induced optical breakdown [19,28]. The seed electrons undergo free carrier absorption by linearly absorbing laser photons through a process known as avalanche ionization [29]. As a result, the seed electrons increase in energy to a value greater than the band gap energy. Through an impact ionization process, valence electrons populate the conduction band, providing more electrons for free carrier absorption [29,30]. Both multiphoton absorption and avalanche ionization increase the electron density to a level where optical breakdown occurs. At optical breakdown, a high-density plasma (electron gas) is formed. (It is important to note that the density of the plasma will depend on the laser pulse energy and repetition rate [31]. For sub-nanojoule laser pulses at high repetition rates, low-density plasma will be formed [31]. This is in contrast to higher density plasmas formed at higher laser powers and lower repetition rates). It is the multiphoton absorption and ionization process that is responsible for laser surgical-ablation [17].

Fs laser pulses provide an ideal tool for manipulating biological material and have significant advantages over longer laser pulse durations (nanoseconds and picoseconds). In long laser pulse ablation processes, optical breakdown is dependent on the density of impurity electrons [32]. As the impurity density changes, the threshold parameters for surgical-ablation either increase or decrease, making the ablation process variable. This is in contrast to fs ablation where the initial seed electrons required for optical breakdown are provided by multiphoton absorption and not by the impurity density of the material. Therefore, in fs laser pulse ablation, threshold parameters can be precisely defined and the ablation process is highly reproducible. Also favoring the use of fs laser pulses is the lower pulse energy required to elicit optical breakdown. Since the degree of collateral damage induced on biological material scales as the pulse energy to the third power (for above threshold energies) [20,33], the lower pulse energy in fs ablation reduces deleterious effects on surrounding material. Mechanical and thermal stresses such as shock waves, cavitation, melting and carbonization are also minimized due to the lower pulse energy funneled into the above processes [17,20].

In this study, when the fs laser pulses were focused to a location near the blastomere-yolk interface, laser surgical-ablation of this region occurred. We observed scarring of the blastomere-surface and formation of cavitation bubbles resulting from the high peak intensity at the laser focal plane. By varying the average laser power and beam dwell time, the spatial extent of blastomere-surface scarring and the radius (or diameter) and decay time of the cavitation bubble could be accurately controlled. This is evidenced in Figure 7 where a decrease in the beam dwell time resulted in both a decrease in the cavitation bubble diameter and the spatial extent of tissue scarring. Specifically, under constant average laser power, ablation using a beam dwell time of 500 ms produced a cavitation bubble that was ~2 times greater than the bubble created using a beam dwell time of 100 ms. This result was found for a single pulsing event of the galvo. Tissue scarring was also reduced, ~5 μm for Figure 7(b) and ~10 μm for Figure 7(d), indicating that decreasing the beam dwell time can reduce the extent of tissue damage. The optical parameters yielding minimal blastomere-surface damage with fast cavitation decay times (within a few seconds) were found to be an average laser power of 40 to 45 mW with a beam dwell time of ≤ 100 ms. Fast cavitation decay times using 40–45 mW average laser power are consistent with the Suppatto *et al*. study, where ablation using similar average laser powers produced cavitation bubbles that decayed within < 5 s [34]. We also addressed whether these laser parameters could be used to form pores in the blastomeres, which would be harnessed as a delivery pathway for introducing exogenous molecules. Suspending dechorionated embryos in the presence of a fluorescent probe, we confirmed the creation of pores by observing blastomere fluorescence. The pores were formed by the previously described nonlinear multiphoton absorption and avalanche mechanism that occurs at the laser focus. Laser-induced pores created near the blastomere-yolk interface were found to be transient, as verified by the absence of cytoplasmic leakage into the extracellular space post laser-manipulation. This observation was in agreement with our previous work on the kinetics of laser-induced pores formed in the membranes of live mammalian cells [25]. Volumetric response plots and membrane integrity assays were used to confirm the transient nature of the laser-induced pores [25].

It was difficult to accurately measure the kinetics of laser-induced transient pores in live zebrafish embryos, due to a low surface-to-volume ratio, low membrane permeabilities, differing osmotic properties of the blastoderm and yolk, and the large size of the yolk and cells [11,35-37]. However, a hypothesis can be made based on our previous work [25]. In Figures 3, 4 and 5 we noticed a decrease in blastomere fluorescence as the beam dwell time was reduced. This decrease was verified by qualitative and quantitative assessments of blastomere fluorescence. The reduction in the observed fluorescence was likely attributed to the kinetics of the pore, where the lifetime of the pore was expected to be the longest for Figures 3(a,b) (largest accumulation of the fluorescent probe) and shortest for Figures 5(a,b) (minimal accumulation of the fluorescent probe). In previously published work, volumetric response plots of porated mammalian cells revealed that the fastest kinetics were on the order of 270 ms [25]. In this work we expected similar pore kinetics, since the mechanism of ablation was identical. While the model systems in this and our previous study on mammalian cells had different membrane structures and compositions, we hypothesized that such differences would not affect the laser-induced pore formation process, as it relies on laser-matter nonlinear interactions (multiphoton absorption and avalanche ionization). Fs ablation is a very efficient process, which has been demonstrated in a variety of materials from metals to biological tissues [19,28,30,38-41]. Provided that the laser parameters for optical breakdown of the model system are at or above the threshold, ablation and subsequent pore formation will always occur, independent of the mechanical properties of the material. Although the membrane composition of the models in this and our previous study differ, it was theorized that the bulk macromolecular structure would be substantially the same. We hypothesized that the 270 ms measured in our previous work represented a valid starting point for estimating the pore duration in this study. However, as the average laser power used in this study was six times lower than that used in our previous work, we expect 270 ms to be a slight overestimate. It was conjectured that the kinetics of the pores would be bounded by a maximum longevity of 270 ms, which would progressively decrease as the beam dwell was reduced. Further work is being conducted on pore dynamics to properly quantify the resealing time for pores created using low average laser powers.

### Delivery of an exogenous fluorescent probe (FITC)

Figures 3, 4 and 5 depict the introduction of FITC into the blastomere cells of developing embryos. All embryos were laser manipulated at 2-cell stage. A total of 6 pores were made in each embryo (3 pores/blastomere). The strongest blastomere fluorescence (53.9 mean intensity/area) was observed with a beam dwell time of 100 ms and a galvo pulse number of 3. Generally, we observed a decrease in the amount of blastomere fluorescence as both the beam dwell time and galvo pulse number were reduced. This was confirmed by both qualitative and quantitative analysis of the blastomere fluorescence intensity. Using a beam dwell time of 50 ms and a galvo pulse rate of 3, as compared to a dwell time of 100 ms and a galvo pulse rate of 1, noticeably brighter fluorescence (29.7 mean intensity/area, 50 ms and 3 pulses of the galvo; 16.9 mean intensity/area, 100 ms and 1 pulse of the galvo) was observed in the blastomere cells (compare Figures 3(c,d) with Figures 4(a,b)). This increased fluorescence was expected, despite the decrease in beam dwell time, as the total laser exposure time for Figures 4(a,b) was 150 ms (50 ms × 3) versus 100 ms (100 ms × 1) for Figures 3(c,d). Longer exposure times would be expected to increase the intracellular accumulation of the fluorescent probe. Therefore, the beam dwell time was not the only parameter contributing to blastomere fluorescence, and the galvo pulsing rate must also be taken into consideration. For laser-induced pores created with multiple pulsing events of the galvo, the competency of the pores was expected to increase, since the first pulsing event would weaken the tensile strength of the membrane, making subsequent pore formation easier. (Competency of the pore was defined as the ability of the pore to deliver exogenous molecules into the developing blastomeres. Laser-induced pores delivering higher intracellular concentrations would have a higher competency. The average laser power, beam dwell time and galvo pulse number all affect the degree of competency.) However, there would be a limitation on the number of galvo events, since each pulsing deposits extra laser energy and increases the cavitation radius. We observed (data not shown) that when the cavitation radius was greater than several micrometers, the competency of the laser-induced pore decreased. We hypothesized that the outward expansion of the cavitation reduces the efficiency of passive diffusion of the fluorescent probe through the pore.

In our experiments both qualitative and quantitative assessments of blastomere fluorescence were made. Fluorescence intensity was evaluated by visually inspecting each embryo under epi-fluorescence using 4×, 10× and 60× magnifications. Quantitative results were obtained through image analysis, where the respective mean fluorescence intensity per unit area of the blastomeres was determined. It is important to mention that FITC is known to be an amine reactive dye. Since proteins were not added to the tank water, no reactivity of the dye with the surface of the blastomere cells was observed. Under high magnification (60×), we confirmed that the fluorescence originated from within the blastomere cells and not from undesirable reactions between the cell membranes and the dye.

Figures 3, 4 and 5 depict the porated embryos post laser-manipulation. The 2-cell stage embryos had developed slightly (1 to 2 cleavage divisions) to 4/8-cell stage between laser treatment and imaging. This development occurred during the time it took to laser manipulate each sequence of embryos, including rinses to remove the fluorescent probe and re-mounting the specimens for imaging. Despite the fact that the embryos in Figures 3, 4 and 5 differed in development by 1 to 2-cleavage divisions, accurate assessments of blastomere fluorescence could still be made. To correct for these small developmental stage differences, a complete image of each whole embryo was taken under a constant exposure time of 1.5 seconds. In this way, the volume occupied by the fluorescent probe, regardless of the developmental stage, should be the same since each cleavage division divided the volume of the cells in half. For instance, in Figure 2(a), the two blastomeres seen originated from the cleavage of a single blastomere cell, and would have approximately the same volume (half that of the original cell). As the cells continued to divide, animal polar views would show increasing cleavage cuts along both the short and long axes of the blastodisc. The blastomere cells on either side of the long axis have approximately the same volume. Synchronous cleavage divisions would continue to reduce the volume of the cells, increasing the number of blastomeres along the short and long axes of the blastodisc while maintaining a constant total volume of all cells.

### Survival and short- and long-term development

Short-term survival was determined by comparing the developmental morphology and hatching rates of laser-manipulated hatched larvae against control larvae. Tracking embryonic development from early cleavage (2-cell) to hatching, we observed no accelerated development or developmental lag in the laser-manipulated hatched larvae relative to the controls. Short-term survival of laser porated embryos reared to 2 dpf was found to be 93% (28/30). SEM mosaics of control and laser-manipulated hatched 2 dpf larvae were compared, providing a more detailed assessment of development. During the fixing and graded alcohol steps, the removal and addition of solutions was performed carefully and each added solution was allowed to equilibrate. This minimized differential shrinkage (see Figures 2(a,b,c)), allowing for accurate larval comparisons. Larvae were mounted on carbon stubs in the dorso-lateral and lateral orientations. We attempted to mount all larvae in the same orientation, however, slight differences in the projected views were expected. Once the larvae were mounted on stubs and partially dried we avoided any manipulation of the larvae to avoid damaging the specimens.

**Figure 12:**
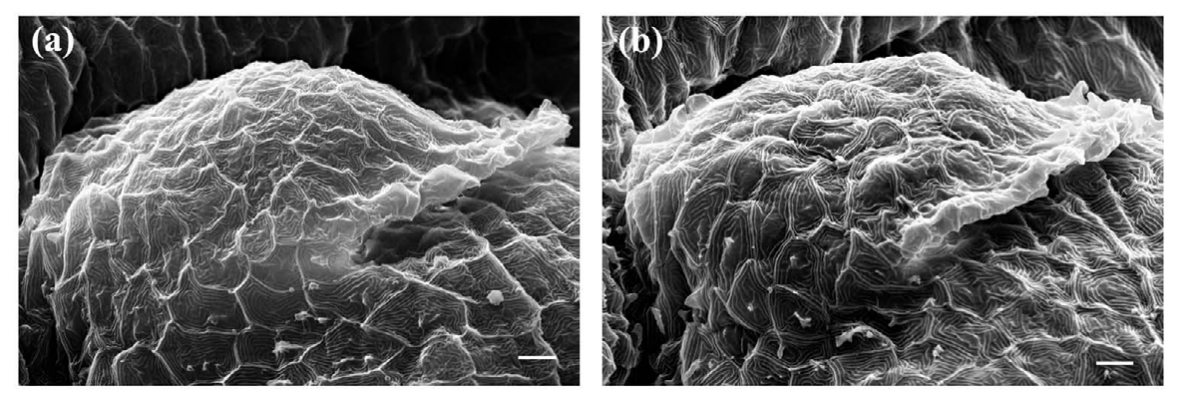
SEM images depicting the pectoral fin bud in larvae reared to 2 dpf. (a) Pectoral fin bud of a laser-manipulated and (b) a control larva at 2 dpf. Scale bar represents 10 μm. Orientation: anterior (left); posterior (right).

As shown in Figure 8, we observed no significant differences in the body plan of the laser-manipulated larva as compared to the control larva. Developmental features such as the caudal fin (CF), dorsal fin (DF), yolk sac extension (YSE), yolk sac (YS) and the olfactory pit (OP) were clearly identifiable in both larvae. In the magnified anterior views of Figure 8, the olfactory pit (OP) was visible in both larvae, with the head of each larva slightly curved over the yolk sac (YS). The pectoral fin buds were difficult to resolve in the laser-manipulated and control samples. At 2 dpf, the pectoral fins began to develop on the lateral sides of each larva just above the yolk sac and slightly posterior to the otic capsule. As the larvae aged, the pectoral fins lifted away from the yolk sac and developed along the lateral extent of the zebrafish body. Figure 12 depicts the pectoral fin buds of a laser-manipulated larva (Figure 12(a)) and a control larva (Figure 12(b)) at 2 dpf. Both fin buds above the yolk sac will develop into the elaborate pectoral fins observed in Figure 10. Comparing the pectoral fin buds in Figure 12 to the pectoral fins in Figure 10, we noticed that the development from immature fin to mature fin occurred normally. No developmental differences were observed in the fin buds of the laser-manipulated larva relative to the control larva. In short, no short-term effects of the laser on the development of the zebrafish embryo were observed.

As previous mentioned, the effects of laser pulses on embryonic development might not be apparent until later developmental stages. To confirm that the laser parameters (45 mW; 100 ms; galvo pulsed 3 times) used in this study did not adversely affect long-term embryonic development, SEM mosaics of control larvae and laser-manipulated larvae at 7 dpf were compared. Figures 9 and 10 show normal development of the laser-manipulated larvae. We see normal development of the whole body plan of both laser-manipulated and control larvae reared to 7 dpf. Figure 10 illustrates key developmental features that were observed in both the laser-manipulated larva and control larva and include the protruding mouth (PM), olfactory pit (OP), eye (E; cornea), otic capsule (OC), otic vesicle (OV), pectoral fin (PF), ventral fin (VF) and notochord (NC). No differences were seen in the placement or patterning of these features between the samples. To conclusively define the application of fs laser pulses as non-invasive, we examined under high magnification the olfactory pit, otic capsule, otic vesicle, posterior forebrain and dorsal midbrain (Figure 11).

**Figure 13:**
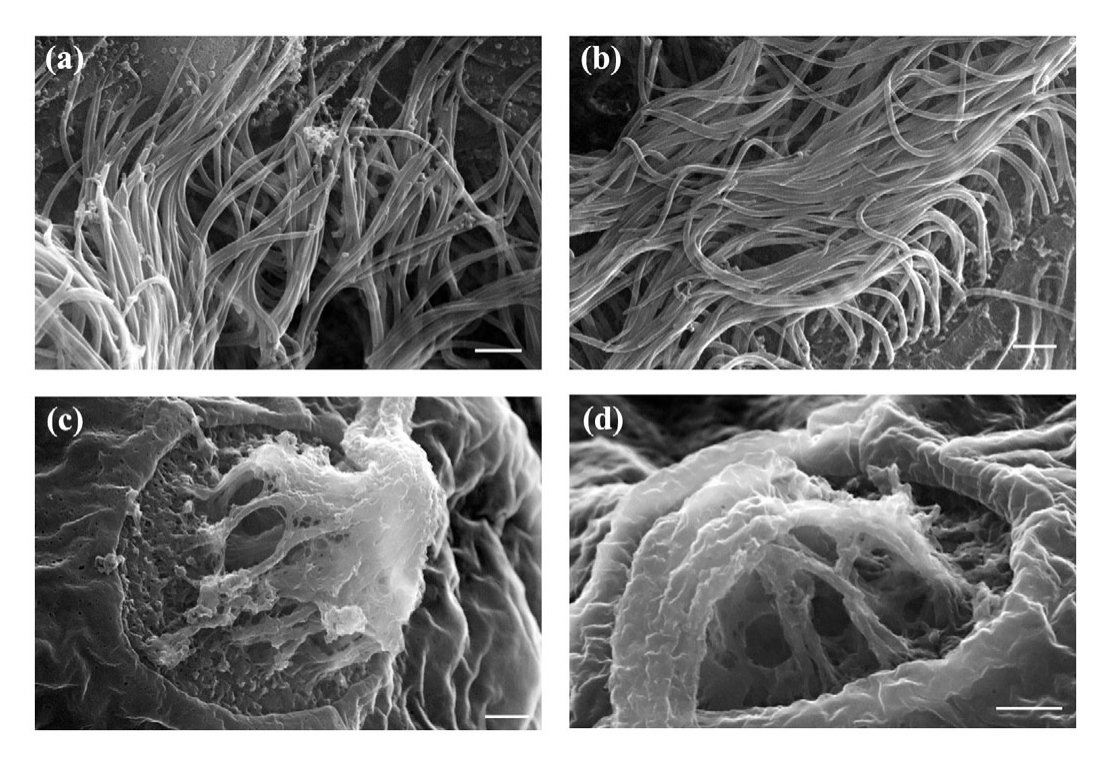
High magnification SEM images of the olfactory pit rim and kinocilia projecting from cristae on the lateral wall into the lumen. (a) Olfactory pit rim in a laser-manipulated and (b) a control larva showing kinocilia covering the inside wall of the olfactory pit. Kinocilia projecting from lateral cristae in (c) a laser-manipulated and (d) a control larva. Scale bars for (a, b, c, d) represent 1 μm.

The zebrafish olfactory organ is responsible for detecting and discriminating thousands of odorants [42]. Courtship depends upon the chemosensitivity of the organ, where specific pheromones act as attractants [42]. Zebrafish rendered anosmic are unable to reproduce [42], indicating that this organ is functionally essential. It has been hypothesized that the organ forms from subepidermal cells, some of which form the olfactory placode [43]. The placode cells differentiate into receptors, supporting cells and ciliated cells [43], which eventually give rise to the olfactory organ. As the olfactory organ develops, the epidermal cells spread, revealing the olfactory pit located on either side of the head posterior to the eye. Figures 11(a) and 11(d) depict the olfactory pits of a laser-manipulated and a control larva reared to 7 dpf, respectively. In both SEM images, epidermal cells surrounded the olfactory pit. Inside the pits, ciliated receptor cells and kinocilia were observed in both laser-manipulated and control larvae. Figures 13(a) and 13(b) show high magnification SEM images of the olfactory pit rim. We noticed that in both larvae, the pit rims were covered by long kinocilia densely covering the inside walls. Together, Figures 11(a,d) and 13(a,b) indicate that the olfactory pits of the laser-manipulated larvae appear to develop similarly to the controls.

Rotational and linear acceleration, gravity and sound are sensed by the zebrafish inner ear and lateral line organ [44,45]. The inner ear forms from the otic placode that originates from the ectoderm [44]. Placode cells give rise to a hollow otic vesicle. As the otic vesicle expands, the lumen of the ear becomes visible [44]. Figure 10 depicts the otic capsule and the otic vesicle in a laser-manipulated and a control larva, where the space inside the otic vesicle is defined as the lumen. As shown in Figure 10, the ear is posterior to the eye and develops on either side of the hindbrain. Figures 11(b) and 11(e) represent magnified SEM images of the zebrafish ear at 7 dpf. Both images are morphologically very similar. On the lateral wall surrounding the lumen, both larvae showed protrusions ending in a long hair-like projection. We believe that these protrusions are cristae with projecting kinocilia. Cristae and associated kinocilia were also found on the anterior, posterior, dorsal and ventral walls of the lumen, however these are difficult to distinguish in Figures 11(b) and 11(e). The projections from the cristae appeared as single hair-like structures in the figures, however, closer examination revealed that several kinocilia were present as expected. Figures 13(c) and 13(d) show magnified SEM images of the lateral cristae observed in Figures 11(b) and 11(e). At the surface of the cristae, numerous kinocilia projected into the lumen. As seen in Figures 13(c) and 13(d), the kinocilia were clumped and bent, unlike in an aqueous environment where kinocilia are polarized. The clumping and bending are believed to be an artifact of the fixing and sputtering required for SEM. Similar protrusions, termed neuromasts, were also observed distributed over the zebrafish body and are likely associated with the lateral line. In control larvae, neuromasts were found anterior to the olfactory pit (Figure 11(d)), at the outer rim of the otic capsule (Figure 11(e)), anterior to the diencephalon (Figures 11(f) and 11(h)) and adjacent to both sides of the optic tectum and diencephalon (Figures 11(f) and 11(h)). Comparing Figures 11(d,e,f,h) with Figures 11(a,b,c,g), neuromast patterning in laser-manipulated larvae was found to be identical to that seen in the controls. Similar to the observations made in Figures 13(c) and 13(d), several kinocilia were present (data not shown). The combined results of Figures 11 and 13 indicate that no long-term effects of the laser on development were observed.

Figures 11(c,g) and 11(f,h) represent SEM images of the neuromeres [46] of the posterior forebrain (diencephalon), dorsal midbrain (optic tectum) and anterior hindbrain (cerebellum) in a laser-manipulated and a control larva. Anterior to the diecephalon is the telencephalon, defining the anterior part of the forebrain [47,48]. Comparing Figures 11(c,g) with 11(f,h), we observed no difference in the morphology and placement of the fore-, mid- and hindbrains. In both the laser-manipulated and control larva, the olfactory pits were slightly anterior to the diencephalon and both the posterior fore- and midbrains were symmetrically placed adjacent to the eye.

## Conclusion

In this study, we have shown that focused fs laser pulses can be used as a tool for manipulating the embryonic cells of the developing zebrafish. Utilizing the ablation mechanism that occurs at the focus, we demonstrated the formation of transient pores near the blastomere-yolk interface. These pores were harnessed as a delivery pathway for introducing exogenous molecules into the blastomere cells of the developing embryo. Delivery is not limited to the intracellular accumulation of a fluorescent probe, but can be extended to the exogenous introduction of cryoprotects and plasmids. (In fact, in a recent article [26] we showed the uptake of conjugated quantum dots and the laser transfection of Simian-CMV-EGFP into the blastomeres of 2-cell to 8/16-cell stage embryos). Comparing the developmental morphology of laser-manipulated larvae with control larvae, we observed no significant differences. The complex patterning of zebrafish organs such as the olfactory organ and ear occurs early in development. These organs appear to originate from placode cells [27,43,44]. If the application of fs laser pulses were invasive, the placode cells would not be expected to develop normally. As a result, morphological changes would be likely seen at later developmental stages. A comparison of viable laser-manipulated larvae with control larvae provides no evidence of abnormal growth, thus making the application of fs laser pulses an extremely powerful, non-invasive tool.

## Methods

### Zebrafish care

25 to 30 adult male and female wildtype zebrafish were kept in 22 L of UV treated reverse osmosis water (subsequently defined as tank water). The temperature of the water was maintained at 28.5 ± 1°C with a pH of 6.8 to 7.2. pH adjustments were made using sodium bicarbonate.

### Breeding and harvesting of embryos

Adult male and female wildtype zebrafish were placed in a breeding tank containing 1 L of tank water. In each breeding tank 3 adult females were paired with 2 adult males. A total of 4 to 5 tanks were set up for simultaneous breeding. A 2 mm wire mesh was placed at the bottom of each breeding tank to protect fallen eggs from being eaten by the adult fish. Breeding tanks were kept on a 10 hr dark/14 hr light cycle. Embryos were harvested at approximately 20 min after the start of the light cycle.

### Dechorionation procedure

A 20 mg/ml stock solution of Pronase (Roche Applied Sciences, IN, USA) was diluted to 10 mg/ml in ddH_2_O. 100 μL was added to a vial containing embryos in 6 ml of tank water. Final concentration of Pronase in solution was 0.16 mg/ml. The vial was periodically agitated to assist in the removal of the proteinaceous membrane (i.e. chorion). After observing fragments of the chorion in solution, the dechorionation process was stopped. The embryos were immediately rinsed 3 to 4 times with fresh tank water, and transferred to a dish containing tank water.

### Preparation of fluorescein isothiocyanate (FITC)

A 0.1 mg/ml stock solution of FITC (Sigma-Aldrich, ON, Canada) was prepared in ddH_2_O. For exogenous delivery, 11 μL of stock FITC was added to the embryos already suspended in 50 to 70 μL tank water, resulting in a final FITC concentration ranging from 0.014 to 0.018 mg/ml.

### Fluorescence imaging

Fluorescence assessment of delivered blastomere-FITC was observed using a standard FITC filter (Chroma Technology Corp., Rockingham, VT). Fluorescence images were captured using the Nikon DS-5M color CCD and ACT2U software (Nikon, Canada) and processed using Photoshop CS2 (Adobe Systems Inc., USA). Epi-fluorescence was detected using a modified 80i upright Nikon microscope, Figure 1. All embryos targeted for poration were exposed to the fluorescent probe for 10 to 15 min. After delivery, embryos were rinsed several times in tank water and imaged 10 to 15 min after poration with a constant imaging area and exposure time of 1.5 sec. The image depicting autofluorescence (non-laser treated embryo) was exposed, rinsed and subsequently imaged using the same conditions stated above.

### Zebrafish development, hatching rates, survival, and morphological integrity

To accurately determine the short- and long-term effects of the applied fs laser pulses, hatching rates, survival and developmental morphologies of control and laser-manipulated embryos were compared. Throughout embryo development, from early cleavage (2-cell) to 2 dpf, both control and laser-manipulated embryos/larvae were visually inspected for differences in hatching rates and for abnormal development (i.e. body plan, curved dorsals, malformation of the body axis and asymmetric yolk sacs). The developmental endpoint for determining survival was taken as 2 dpf. Laser-manipulated larvae that exhibited either delayed or accelerated growth or abnormal development in comparison to control larvae were considered compromised and non-viable. Embryo survival was determined based on normal hatching rates and morphology. Morphological assessments were performed by comparing LM images and SEM mosaics of control larvae and laser-manipulated larvae. Long-term laser effects on zebrafish development were determined by rearing hatched larvae until 7 dpf. SEM mosaics of control and laser-manipulated larvae were examined with an emphasis on comparing developmental features such as the olfactory pit, otic capsule, otic vesicle, posterior forebrain, dorsal midbrain and anterior hindbrain, as well as the whole body plan.

### Fixing and mounting

Early to mid cleavage stage embryos (2-cell to 4/8-cell) and 2 and 7 dpf hatched larvae were fixed and mounted for SEM imaging. Embryos and larvae were incubated for 2 1/2 hours in 2% glutaraldehyde (Sigma-Aldrich, ON, Canada) or 10% formaldehyde (Fisher Scientific, ON, Canada), both prepared in ddH_2_O. Post-incubation, the embryos and larvae were washed 3 to 4 times in ddH_2_O with an interval of 15 min between each wash. After complete removal of glutaraldehyde/formaldehyde, graded alcohol steps were performed using 30, 50, 70, 90 and 100% ethanol (EtOH). The embryos and larvae were bathed for 15 min in 30, 50 and 70% EtOH, for 5 min in 90% EtOH and for 10 min in 100% EtOH. To remove remaining ddH_2_O, the samples were washed 3 to 4 times for 10 min in 100% EtOH. Before mounting the embryos and larvae on SEM stubs, the 100% EtOH was gradually replaced with hexamethlydisilazane (HMDS) (Fisher Scientific, ON, Canada). Solutions of 75:25, 50:50 and 25:75% EtOH:HMDS were prepared, and the samples were incubated in each for 10 min. Remaining EtOH was removed by washing the embryos and larvae 3 to 4 times in 100% HMDS for 10 min. The embryos and larvae were either incubated in 100% HMDS overnight or immediately mounted on carbon tape SEM stubs. Each stub was allowed to dry for 20 to 30 min before being coated with palladium.

### Sputtering and SEM imaging

Embryos and larvae were coated with palladium using the Hummer 6.2 Sputtering System (Anatech Ltd., Hayward, CA). The vacuum of the sputtering chamber was allowed to reach 30 millitorr before being purged with Argon gas. The chamber was purged until the vacuum reached 200 millitorr, after which the vacuum was adjusted to 55 to 65 millitor. The sample stage was rotated and a plasma discharge was maintained for coating the specimens with palladium. Specimens were coated for 90 to 150 sec. All samples were stored in a desiccator before being imaged. SEM imaging was performed using the XL 30 Series Philips ESEM LaB_6 _and the Leo 1430 SEM. The accelerating voltage ranged from 10 to 30 kV.

### Optical setup, laser poration and exogenous delivery

Sub-10 fs laser pulses were generated from a Kerr lens modelocked titanium sapphire laser oscillator with a centre wavelength of 800 nm and a pulse repetition rate of 80 MHz, Figure 1. Early to mid cleavage stage (2-cell to 4/8-cell) embryos were placed on a motorized *x*-*y*-*z *stage, with an *x*-*y *translation speed of 1 mm/sec and a *z*-focus step resolution of 50 nm, Figure 1. Both the average laser power and beam dwell time were varied from 25 to 50 mW (the average laser powers coupled into the microscope objective) and from 5 to 500 ms, respectively, to determine the minimal average laser power and beam dwell time required for surgical-ablation. Based on visual observation of the laser-manipulated embryos, optimal laser parameters for transient pore formation and delivery of an exogenous fluorescent probe were defined. The transient pore was formed by focusing fs laser pulses with a 1.0 NA 60× water immersion microscope objective to a spot size of ~800 nm. A total of six pores (three per blastomere) were created in each embryo near the blastomere-yolk interface with a maximum of 2 to 4 blastomeres laser treated.

### Image analysis

ImageJ analysis software (National Institute of Health) was used to quantify the mean fluorescence intensity per unit area originating from the blastomere cells. The analyzed fluorescence images were captured using the Nikon DS-5M color CCD. This CCD has a sensor size of 8.8 mm (W) × 6.6 mm (H) with a pixel size of 3.4 μm and an effective resolution of 5 million pixels. Images captured with the DS-5M have a 4:3 aspect ratio with the color pixels arranged in a Bayer grid filter fashion. To accurately determine the mean fluorescence intensity the image scale in ImageJ was set to 294.12 pixels/mm with an aspect ratio of 4:3 (1.333) in accordance with the CCD specifications. All analyzed images were of the same dimensions. For each image, a blastomere area was defined in the respective brightfield image and this area was then superimposed onto the fluorescent image of the same embryo. The mean fluorescence intensity was measured in the green channel of the CCD. Mean fluorescence intensity was divided by the blastomere area to give the mean fluorescence intensity per unit area originating from the blastomere cells.

## Authors' contributions

VK carried out all experimental work and design of the reported study. The presented work is in partial fulfillment of VK's PhD thesis. AE provided the infrastructure for performing the experiments, read the manuscript and offered valuable comments. All authors have read and approved the final manuscript.

## Supplementary Material

Additional file 1 Video demonstrating surgical-ablation of a 2-cell stage chorionated embryo. First video sequence depicts the 2-cell stage chorionated embryo at 4× and 60× magnification. Second video sequence shows ablation using an average laser power of 45 mW with a beam dwell time of 100 ms and the galvo pulsed a total of 3 times. Third video sequence depicts ablation of the same embryo at a different location using an average laser power of 45 mW with a beam dwell time of 500 ms and a total of 2 galvo pulsings. Ablation was performed near the blastomere-yolk interface using a 1.0 NA water immersion microscope objective. Click here for file

Additional file 2 Larva laser-manipulated at the 2/4-cell stage and subsequently reared to 5 dpf. Live video shows the top view of the developed larva. Click here for file

## References

[B1] Vogel G (2000). Zebrafish Earns Its Stripes in Genetic Screents. Science.

[B2] Sar AM, Appelmelk BJ, Vandenbroucke-Grauls CMJE, Bitter W (2004). A star with stripes: zebrafish as an infection model. Trends Microbiol.

[B3] Weinberg ES, Hennig W (1992). Analysis of Early Development in the Zebrafish Embryo. Early Embryonic Development of Animals.

[B4] Barut BA, Zon LI (2000). Realizing the potential of zebrafish as a model for human disease. Physiol Genomics.

[B5] Warren KS (2000). The genetic basis of cardiac function: dissection by zebrafish (Danio rerio) screens. Phil Trans R Soc Lond B.

[B6] Dooley K, Zon LI (2000). Zebrafish: a model system for the study of human disease. COGD.

[B7] Hill AJ, Teraoka H, Heideman W, Peterson RE (2005). REVIEW: Zebrafish as a Model Vetebrate for Investigating Chemical Toxicity. Toxicol Sci.

[B8] Jagadeeswaran P, Sheenhan JP (1999). Analysis of Blood Coagulation in the Zebrafish. Blood Cells Mol Dis.

[B9] Nasevicius A (2000). Effective targeted gene 'knockdown' in zebrafish. Nat Genet.

[B10] Thisse C, Zon LI (2002). Organogenesis-Heart and Blood Formation from the Zebrafish Point of View. Science.

[B11] Hagedorn M, Hsu E, Kleinhans FW, Wildt DE (1997). New Approaches for Studying the Permeability of Fish Embryos: Toward Successful Cryopreservation. Cryobiology.

[B12] Hagedorn M, Kleinhans FW, Freitas R, Liu J, Hsu EW, Wildt DE, Rall WF (1997). Water Distribution and Permeability of Zebrafish Embryos, Brachydanio rerio. J Exp Zool.

[B13] Roeser T, Baier H (2003). Visumotor Behaviors in Larval Zebrafish after GFP-Guided Laser Ablation of the Optic Tectum. J Neurosci.

[B14] Yamaguchi M, Yoshimoto E, Kondo S (2007). Pattern regulation in the stripe of zebrafish suggests an underlying dynamic and autonomous mechanism. PNAS.

[B15] Yang CT, Sengelmann RD, Johnson SL (2004). Larval Melanocyte Regeneration Following Laser Ablation in Zebrafish. J Invest Dermatol.

[B16] Jones JE, Corwin JT (1996). Regeneration of Sensory Cells after Laser Ablation in the Lateral Line System: Hair Cell Lineage and Macrophage Behavior Revealed by Time-Lapse Video Microscopy. J Neurosci.

[B17] Niemz M, Springer  (2002). Laser-Tissue Interactions: Fundamentals and Applications. Biological And Medical Physics.

[B18] Noack J (1999). Laser-Induced Plasma Formation in Water at Nanosecond to Femtosecond Time Scales: Calculation of Thresholds, Absorption Coefficients, and Energy Density. IEEE J Quantum Elect.

[B19] Oraevsky AA, Da Silva LB, Rubenchik AM, Feit MD, Glinsky ME, Perry MD, Mammini BM, Small W, Stuart BC (1996). Plasma Mediated Ablation of Biological Tissues with Nanosecond-to-Femtosecond Laser Pulses: Relative Role of Linear and Nonlinear Absorption. IEEE J Quantum Elect.

[B20] Vogel A, Busch S, Jungnickel K, Birngruber R (1994). Mechanisms of Intraocular Photodisruption With Picosecond and Nanosecond Laser Pulses. Laser Surg Med.

[B21] Kohli V, Acker JP, Elezzabi AY (2005). Cell nanosurgery using ultrashort (femtosecond) laser pulses: Applications to membrane surgery and cell isolation. Laser Surg Med.

[B22] Tirlapur UK, Konig K (2002). Femtosecond near-infrared laser pulses as a versatile non-invasive tool for intra-tissue nanoprocessing in plants without compromising viability. Plant J.

[B23] Heisterkamp A, Maxwell IZ, Mazur E, Underwood JM, Nikerson JA, Kumar S, Ingber DE (2005). Pulse energy dependence of subcellular dissection by femtosecond laser pulses. Opt Express.

[B24] Konig K, Riemann I, Fritzsche W (2001). Nanodissection of human chromosomes with near-infrared femtosecond laser pulses. Opt Lett.

[B25] Kohli V, Acker JP, Elezzabi AY (2005). Reversible permeabilization using high-intensity femtosecond laser pulses: Applications to biopreservation. Biotechnol Bioeng.

[B26] Kohli V, Robles V, Cancela ML, Acker JP, Waskiewicz AJ, Elezzabi AY (2007). An alternative method for delivering exogenous material into developing zebrafish embryos.. Biotechnology and Bioengineering.

[B27] Kimmel CB, Ballard WIW, Kimmel SR, Ulmann B, Schilling TF (1995). Stages of Embryonic Development of the Zebrafish. Developmental Dynamics.

[B28] Loesel FH (1998). Non-thermal ablation of neural tissue with femtosecond laser pulses. Appl Phys B.

[B29] Schaffer CB, Brodeur A, Mazur E (2001). Laser-induced breakdown and damage in bulk transparent materials induced by tightly focused femtosecond laser pulses. Meas Sci Technol.

[B30] Gamaly EG, Rode AV, Luther-Davies B (2002). Ablation of solids by femtosecond lasers: Ablation mechanism and ablation thresholds for metals and dielectrics. Phys Plasmas.

[B31] Vogel A, Noack J, Huttman G, Paltauf G (2005). Mechanisms of femtosecond laser nanosurgery of cells and tissues. Appl Phys B.

[B32] Schaffer CB, Garcia JF, Mazur E (2003). Bulk heating of transparent materials using a high-repetition-rate femtosecond laser. Appl Phys A.

[B33] Zysset B, Fujimoto JG, Puliafito CA, Birngruber R, Deutsch TF (1989). Picosecond Optical Breakdown: Tissue Effects and Reduction of Collateral Damage. Laser Surg Med.

[B34] Supatto W, Debarre D, Moulia B, Brouzes E, Martin JL, Farge E, Beaurepaire E (2005). In vivo modulation of morphogenetic movements in Drosophila embryos with femtosecond laser pulses. PNAS.

[B35] Janik M, Kleinhans FW, Hagedorn M (2000). Overcoming a Permeability Barrier by Microinjecting Cryoprotectants into Zebrafish Embryos (Brachydanio rerio). Cryobiology.

[B36] Liu XH, Zhang T, Rawson DM (1998). Feasibility of vitrification of zebrafish (Danio rerio) embryos using methanol. Cryo Letters.

[B37] Liu XH, Zhang T, Rawson DM (1999). The Effect of Partial Removal of  Yolk on the Chilling Sensitivity of Zebrafish (Danio Rerio) Embryos. Cryobiology.

[B38] Neev J, Da Silva LB, Feit MD, Perry MD, Rubenchik AM, Stuart BC (1996). Ultrashort Pulse Lasers for Hard Tissue Ablation. IEEE Journal of Selected Topics in Quantum Electronics.

[B39] Chichkov BN, Momma C, Nolte S, Alvensleben FV, Tunnerman T (1996). Femtosecond, picosecond and nanosecond laser ablation of solids. Appl Phys A.

[B40] Lenzner M, Kruger J, Sartania S, cheng Z, Spielmann C, Mourou G, Kautek W, Krausz F (1998). Femtosecond Optical Breakdown in Dielectrics. Phys Rev Lett.

[B41] Perez D, Lewis LJ (2002). Ablation of Solids under Femtosecond Laser Pulses. Phys Rev Lett.

[B42] Michel WC, Lubomudrov LM (1995). Specificity and sensitivity of the olfactory organ of the zebrafish, Danio rerio. J Comp Physiol A.

[B43] Hansen A, Zeiske E (2004). Development of the olfactory organ in the zebrafish, Brachydanio rerio. J Comp Neu.

[B44] Haddon C, Lewis J (1996). Early Ear Development in the Embryo of the Zebrafish, Danio rerio. J Comp Neu.

[B45] Nicolson T (2005). The Genetics of Hearing and Balance in Zebrafish. Annu Rev Genet.

[B46] Kimmel CB (1993). Patterning the brain of the zebrafish embryo. Annu Rev Neurosci.

[B47] Solnica-Krezel L (2002). Pattern formation in zebrafish. Results and problems in cell differentiation.

[B48] Haffter P, Granato M, Brand M, Mullins MC, Hammerschmidt M, Kane DA, Odenthal J, Eeden FJM, Jiang YJ, Heisenberg CP, Kelsh RN, Furutani-Seiki M, Vogelsang E, Beuchle D, Schach U, Fabian C, Nusslein-Volhard C (1996). The identification of genes with unique and essential functions in the development of the zebrafish, Danio rerio. Development.

